# The role of pharmacists in complementary and alternative medicine in Lebanon: users’ perspectives

**DOI:** 10.1186/s12906-021-03256-8

**Published:** 2021-03-02

**Authors:** Mohamad Ali Hijazi, Hibeh Shatila, Zeina Omeich, Abdalla El-Lakany, Maha Aboul Ela, Farah Naja

**Affiliations:** 1 Faculty of Pharmacy, Department of Pharmaceutical Sciences, Beirut Arab University, P.O. Box: 11 5020, Beirut, Lebanon; 2 Nutrition and Food Sciences Department, Faculty of Agriculture and Food Sciences, American University of Beirut, Beirut, Lebanon; 3 Department of Clinical Nutrition and Dietetics, College of Health Sciences, Research Institute of Medical & Health Sciences (RIMHS), University of Sharjah, Sharjah, United Arab Emirates; 4 Faculty of Agriculture and Food Sciences, American University of Beirut, Beirut, Lebanon

**Keywords:** Pharmacist, Customers, Expectation, Satisfaction, Complementary alternative medicine, Lebanon

## Abstract

**Background:**

Customers’ expectations and satisfaction are critical to ensure a more effective role of the community pharmacists in promoting the safe use of Complementary and Alternative Medicine (CAM). The aim of this study is to examine the perceptions and practices of customers buying their CAM products from pharmacies and explore their satisfaction with CAM-related services offered by the community pharmacists in Lebanon.

**Methods:**

A national cross-sectional study was conducted among users of CAM (age > =18 years) who obtained their CAM from community pharmacies in Lebanon (*n* = 832). Within the proximity of the pharmacy, subjects were invited to complete a multi-component questionnaire. The latter consisted of four sections related to CAM: general beliefs, perception of pharmacists’ role, practices, satisfaction with services offered by the pharmacists. In addition, the questionnaire included questions about sociodemographic characteristics of participants.

**Results:**

The majority of participants agreed to an active role of the pharmacists’ in guiding CAM use, however over half of the participants (61.3%) did not agree that the pharmacist is more knowledgeable in this field than other healthcare providers. As for practices, one in two surveyed customers (47%) did not always give feedback to their pharmacists about the outcomes after using CAM, 20% did not often ask the pharmacists about the safe and effective mode of use of the products and 28.1% did not discuss their medical history. For services offered by the pharmacist, the majority of participants reported receiving good education about the CAM product (87.1%), its side effects (87.1%) and mode of use (93.4%), while significant proportions of participants reported that pharmacists were not asking questions about their medical history before dispensing CAM products (22%) nor were they providing information on CAM-drug interactions (30%).

**Conclusions:**

The results of this study highlighted important gaps between the perceptions of customers and the services they received from the pharmacists about CAM use. These findings could be used by concerned stakeholders, including public health authorities and educational bodies, to develop evidence-based interventions aimed at promoting the role of pharmacists in ensuring a safe and effective CAM use Lebanon.

**Supplementary Information:**

The online version contains supplementary material available at 10.1186/s12906-021-03256-8.

## Background

Complementary and alternative medicine (CAM) refers to various therapies that are not considered part of conventional medicine. CAM can be divided into two major areas 1) natural products which are also called dietary supplements including herbs, probiotics, vitamins and minerals, and 2) mind-body therapies such as meditation, yoga, chiropractic, acupuncture and hypnotherapy [1]. In the last decade, the use of CAM has increased steadily in both developed and developing countries, whereby CAM global market exceeded US$100 billion during year 2017, with natural products making up the most widely used category of CAM therapies [2–5]. Such an increase in the use of CAM has been attributed to many reasons including the dissatisfaction with conventional medicine, unavoidable side effects of many main stream treatments and/or the financial burden associated with their use [6]. Furthermore, the desire to be involved in the decision-making process related to one’s health has also been suggested as a main driver to pull people towards the use of CAM [6–8].

In line with the global trend, the Middle East and North Africa (MENA) region is witnessing a growing market for CAM products [9–13]. In Lebanon, a small country of the MENA, one-third of Lebanese adults (29.87%) are CAM users [6]. Higher prevalence rates were reported among patients with chronic diseases in the country: infertility (41%), lung cancer (41%), and HIV and AIDS (46.6%) [14–16]. Disconcerting traits of CAM use in Lebanon are the low rate of disclosure to the treating physicians coupled to poor regulatory frameworks of CAM use and CAM market [6, 15, 17–20]. Furthermore, a common belief among many CAM users is that CAM products are natural and therefore safe, however, many do not take into consideration the issues related to the toxicity, proper dosage, the risk of contamination and the potential interaction with synthetic and other natural drugs [21, 22]. Such a situation raises concerns about CAM safety, efficacy and impact on the users’ health and wellbeing.

Among health care professionals, community pharmacists are often considered the first contact point between the patient and the healthcare system, perceived as accessible and trustworthy, and have frequent contact with patients due to prescription dispensing schedules [23]. Ideally situated within the community, they can be involved in a broad range of health promotion campaigns and services, including the promotion of safe and effective CAM use. Recently, a national study on the beliefs, practices and knowledge of community pharmacists regarding CAM in Lebanon showed that pharmacists generally have positive beliefs towards CAM use and are greatly involved in dispensing CAM, despite a few gaps in their practices and knowledge which were found to be mainly related to counseling patients and raising awareness of CAM-drug interactions [24]. Such an active role of pharmacists in CAM has also been observed in other countries. For instance, in Australia, a survey of 1500 pharmacists showed that most participants were aware of their important role as educators of CAM and viewed CAM as an integral part of their practice [25]. Similar findings were reported in Palestine, whereby pharmacists agreed to their central role in dispensing CAM and also in educating the patients about its safe use [26]. A systematic review of studies addressing pharmacists and CAM found that ensuring the safety of CAM, documenting its use, reporting adverse effects, educating patients, and collaborating with other health care professional were among the main responsibilities of the pharmacists *vis-à-vis* CAM [27].

An important factor that could lead to a more effective role of pharmacists in the field of CAM use is a clear understanding of the customers’ needs, expectations and satisfaction with the pharmacists’ services. Pharmacists work within a customer-pharmacists professional framework which is based on trust, understanding and shared decision making [28, 29]. However, pharmacists and patients both have specific and significant roles and responsibilities within this framework [30]. The professional value of a pharmacist and the successful implementation of the pharmaceutical care framework depends on how the customers perceive and understand the pharmacist’s professional role especially with respect to direct customer care activities [31]. The customers’ opinions and views of pharmacists’ performance are critical to improving the quality services, enhancing communication and balancing expectations [32]. In this context, the characterization of misconceptions and gaps of the general public regarding the roles and responsibilities of the pharmacists will help in improving the quality of services provided by pharmacists as well as the customer-pharmacist relationship, leading ultimately to a more safe and effective use of CAM [33]. Hence, the main objective of this study is to examine the beliefs, perceptions and practices of customers buying their CAM products from pharmacies in Lebanon, specifically their needs and expectations. Another objective of this study is to assess the satisfaction level of these customers with services provided by community pharmacists as related to CAM products.

## Methods

### Data collection

A national cross-sectional study was conducted among users of CAM who obtained their CAM from community pharmacies in Lebanon from January to July, 2019. A list of all the community pharmacies and their locations in the different governorates was obtained from the Order of Pharmacists in Lebanon (OPL). Pharmacies were selected from this list using a stratified random sampling technique. The strata were the Lebanese governorates. The number of pharmacies was proportional to the number of pharmacies in each stratum. From each pharmacy, one participant (CAM-user) was invited and recruited in the study. The inclusion criteria were 1) age of 18 years or older, 2) buying CAM products for their own use from the selected pharmacy and 3) conversant in either Arabic or English languages. At the pharmacy, the field researcher approached individuals and asked them whether they have bought a CAM product from the pharmacy. In case they did, the field researcher introduced the study and assured the participants that any information collected would be confidential, strictly used for research purposes and that their answers will not affect the services they are receiving at the pharmacy. Subjects were informed that they are free not to answer any of the questions and to withdraw at any point during the interview. After obtaining a signed written consent, a face-to-face interview was conducted within the proximity to the pharmacy. Each interview lasted 10–15 min and required completion of a multicomponent questionnaire. The field researchers were extensively trained in conducting interviews and administering the questionnaire prior to initiation of data collection. The study protocol was approved by the Institutional Review Board at the Beirut Arab University under the protocol number 2019H-0052-P-R-0342.

The questionnaire used for data collection consisted of five main sections. The first section was designed to collect general information about the individuals and included questions on sociodemographic characteristics, CAM use, how the subject was introduced to CAM as well as if using CAM as alternative or complementary to mainstream medicine. In this section, the subject was also asked if he/she suffers from any disease. The second section of the questionnaire included five questions related to the participant’s general beliefs about CAM and its use. The third section of the questionnaire addressed the perception of pharmacists’ role in relation to CAM among study participants. The fourth section included questions on practices of the study participants regarding CAM in the pharmacies. In sections two, three, and four, for each statement, participants marked their degree of agreement on a five-point Likert scale (1 = strongly disagree, 2 = disagree, 3 = neutral, 4 = agree and 5 = strongly agree and 1 = always, 2 = often, 3 = sometimes, 4 = rarely and 5 = no). The last section inquired about the CAM-related services offered by the pharmacists to the subjects and their corresponding satisfaction. The latter was assessed using a Likert rate scale with one representing strongly satisfied and five strongly dissatisfied. The questionnaire was developed by an expert panel consisted of pharmacists, nutritionist, and an epidemiologist, as well as by an extensive review of relevant literature [34–39]. The expert panel ensured the face and content validity of the questionnaire by aligning the questions with the study objectives, examining their relevance in assessing the concepts of perceptions, practices and satisfaction within the context of this study and making sure that all the important elements of these main constructs are adequately addressed. The significance and the wording of the questions were scrutinized for their relevance and cultural acceptability. The questionnaire was originally written in English, translated to Arabic, and then back translated to English. The original and back-translated English versions of the questionnaire were compared to ensure parallel form reliability. The questionnaire was pilot tested on 15 randomly selected individuals to assure the clarity of the questions. The results of the pilot test were not included in the analysis for this study. A copy of the questionnaire is available in Additional file 1: appendix A.

### Statistical analysis

The collected data were coded, entered and analyzed using the Statistical Package for Social Sciences (SPSS) software version 25.0 for windows. For the summary of the data, descriptive statistics consisting of frequency and proportions were used. The associations of sociodemographic factors and CAM use characteristics of participants with their beliefs, perceptions and practices towards the role of pharmacists in CAM were examined using simple and multiple linear regressions. For this purpose, using the answers of the participants, overall scores were created for each of the followings: beliefs, perceptions and practices. For the overall belief score, subjects received a score of either 0, 1, 2, 3, 4, or 5 corresponding to the following answers: strongly disagree, disagree, neutral, agree and strongly agree respectively. Scoring was reversed for statements with negative beliefs towards CAM. Therefore, the higher the score, the more positive is the belief with regards to the role of pharmacist in CAM. A similar protocol was used to calculate an overall score for the perception of the pharmacists’ role in relation to CAM. As for practices, the scores of 0, 1, 2, 3, 4, or 5 corresponded to the following answers: always, often, sometimes, rarely and never. In the regression analyses, the scores were considered dependent variables while sociodemographic factors and CAM use characteristics of participants were independent variables. Variables that were significantly associated with the outcome (*p*-value< 0.2) in the simple regression analyses were entered in the multiple regression model. Statistical significance was set at *p* < 0.05.

## Results

Of the 1000 subjects approached, 832 agreed to participate, response rate (83.2%). The main reasons for refusal to participate were lack of time and interest in the study. The distribution of participants in the study is proportional to the distribution of pharmacies in the governorate (Table 1) with exception to Beqaa. During the time of data collection, the security situation in the Beqaa governorate was not stable and had an ongoing risk of sporadic military violence.

**Table 1:** Distribution of study participants across governorates in this study in *n* = 832

	Participants in the study n(%)	Pharmacies in Lebanon n(%)
**Beirut and mount Lebanon**	472(56.7)	1549(50.9)
**South and Nabatiyeh**	205(24.6)	576(18.9)
**North**	126(15.1)	436(14.3)
**Beqaa**	29(3.5)	482(15.8)
**Total**	**832**	**3043**

The socio-demographic and CAM use characteristics of the overall study population are presented in Table 2. The age of the study population was almost evenly distributed across four age groups (18–25, 26–33, 34–50 and > 51 years). Fifty-seven percent of the participants were females.

**Table 2:** Socio-demographic and CAM use characteristics of study participants. (*n* = 832)

	Frequency	Percentage
**Age range (years)**		
18–25	247	29.7
26–33	176	21.2
34–50	255	30.6
≥ 51	154	18.5
**Gender**		
Male	360	43.3
Female	472	56.7
**Employments status**		
Self-employed	106	13.4
Employee	354	44.6
Not working/student	333	42.0
**Highest educational level attained**		
No education /Primary school	114	13.9
High school	173	21.1
Bachelors /Higher degrees	532	65.0
**Why are you using CAM?**		
Chronic disease	153	19.0
Minor illness	310	38.4
Wellbeing	344	42.6
**Are you using CAM as:**		
Complementary medicine	341	43.5
Alternative Medicine	443	56.5
**What type of CAM products are you using?**		
Herbal	438	54.5
Supplements	366	45.5
**How frequent are you using CAM**		
Rarely (≤1/month)	27	3.3
Often (> = 1 montlhy and ≤ 1 /day	493	60.3
Frequently (> 1/day)	297	36.4
**How were you introduced to CAM?**		
Physician	322	39.0
Pharamcists	319	38.6
Herbalist	34	4.1
Self-decision	99	12.0
From media	52	6.3
**Do you suffer from any disease**		
Yes	215	26.4
No	600	73.6

Table 3 describes the general beliefs with regard to CAM and CAM use among the study participants. The majority of the participants (84.6%) were strongly agreeing or agreeing that CAM products are effective and that they have less side effect than conventional medicine (76.3%). When asked about their beliefs regarding the quality of CAM products sold in the Lebanese market, 53.4% of the participants strongly agreed or agreed that they are of good quality. More than half of the study population (54.8%) believed (strongly agreeing or agreeing) that CAM should be used for minor diseases.

**Table 3:** General beliefs with regards to CAM and CAM use among study participants. (*n* = 832)^*^

	Strongly agree	Agree	Neutral	Disagree	Strongly disagree
Do you belief that CAM products are effective	352(42.3)	352(42.3)	92(11.1)	19(2.3)	17(2.0)
Do you belief that CAM products have less side effect than conventional medicines	307(36.9)	327(39.4)	125(15.0)	46(5.5)	26(3.1)
Do you think that CAM products available in the Lebanese market are of good quality	160(19.3)	283(34.1)	235(28.3)	110(13.3)	42(5.1)
Do you think that CAM should be used only for minor diseases	203 (24.4)	253(30.4)	208(25.0)	114(13.7)	53(6.4)
Do you think that CAM can replace medications for chronic and serious diseases	109(13.1)	125(15.0)	186(22.4)	206(24.8)	205(24.7)

^*^Values in this table represent n (%)

Half of the participants (49.5%) strongly disagreed or disagreed that CAM can replace medications for chronic diseases and serious illnesses.

Perception of the pharmacist’s role towards CAM among study participants is displayed in Table 4. Almost all of the participants (93.6%) agreed or strongly agreed that it is the pharmacist responsibility to inform customers on how to use CAM products and to warn them of possible side effects. In addition, 97.2% agreed or strongly agreed that the pharmacist should answer CAM-related question. Eighty-eight percent of the participants strongly agreed or agreed that they can trust the information given by the pharmacists on the use of CAM products.

**Table 4:** Perception of pharmacists’ role in relation to CAM among study participants. (*n* = 832)^*^

	Strongly agree	Agree	Neutral	Disagree	Strongly disagree
Do you think that the pharmacist should let you know how to use CAM products and warn you for any possible side effects	560(67.3)	219(26.3)	25(3.0)	7(0.8)	21(2.5)
Do you think that pharmacist should answer your CAM related questions	556(67.0)	223(26.9)	23(2.8)	13(1.6)	15(1.8)
Do you trust the pharmacist for the information on the use of CAM products	434(52.2)	297(35.7)	71(8.5)	17(2.0)	12(1.4)
Do you think that pharmacists should advice customerson general health issues other than about CAM products	383(46.3)	283(34.2)	106(12.8)	35(4.2)	20(2.4)
Do you think that pharmacists are more expert in CAM products than other healthcare providers?	172(21.4)	136(17.0)	166(20.7)	143(17.8)	185(23.1)

^*^Values in this table represent n (%)

Table 5 describes the practices of study participants regarding CAM in the pharmacies. Seventy-three percent of the study population reported always or often buying CAM products from pharmacies and 78.7% always/often ask their pharmacists about the effect and safe use of CAM products. Almost half (47.3%) of the study participants did not regularly give their pharmacists feedback about the outcome after CAM usage. If experienced adverse reaction related to CAM, 77.4% reported always/often giving feedback to their pharmacists. Most of the participants (72.1%) always/often inform their pharmacists of their health status and medications usage before taking CAM products.

**Table 5:** Practices of study participants regarding CAM in the pharmacies. (n = 832)^*^

	n(%)				
	Always	Often	Sometimes	Rarely	No
How frequently do you buy your CAM products from the pharmacy?	501(60.2)	103(12.4)	64(7.7)	79(9.5)	85(10.2)
Do you ask your pharmacist about the effective and safe use of the products	375(45.1)	279(33.6)	94(11.3)	44(5.3)	39(4.7)
Do you give your pharmacist feedback about the outcome after you use CAM	217(26.2)	222(26.8)	180(21.7)	85(10.3)	125(15.1)
Do you give your pharmacist feedback if you suffered from any adverse reaction related to CAM products use	381(45.8)	263(31.6)	91(10.9)	51(6.1)	46(5.5)
Do you discuss your health status (diseases) and medications taken with your pharmacists before taking CAM products?	332(39.9)	268(32.2)	132(15.9)	48(5.8)	52(6.3)

^*^Values in this table represent n (%)

The frequency of services offered by pharmacists with regards to CAM and their corresponding satisfaction among study participants is displayed in Table 6. When asked, “did your pharmacists give you information on CAM products”, 87.1% answered yes, and of those who received this service, 89.3% reported to be strongly satisfied/satisfied. Similar distributions were noted for the rest of the services, including examining the medical history prior to dispensing CAM, explaining the mode of CAM use, spending enough time with the customer, offering information in an understandable manner and providing information on CAM-drug interactions.

**Table 6:** Services offered by pharmacists with regards to CAM and their corresponding satisfaction among study participants. (n = 832)^*^

		n(%)				
	Received the service	Of those who received the service				
		Strongly satisfied	Satisfied	Neutral	Dissatisfied	Strongly dissatisfied
Did your pharmacist give you information on the side effect of CAM (Yes = 725)	725(87.1)	342(47.2)	305(42.1)	43(5.9)	6(0.8)	0(0.0)
Did your pharmacist ask you questions about your medical history before dispensing CAM (like disease history, medications, allergy etc.)? (Yes = 651)	651(78.2)	327(50.2)	263(40.4)	33(5.1)	3(0.5)	1(0.2)
Did your pharmacist tell you how to use CAM products (dose, timing, duration, etc.) (Yes = 777)	777(93.4)	411(52.9)	301(38.7)	29(3.7)	2(0.3)	1(0.1)
Did your pharmacist spend enough time with you when you ask about CAM product? (Yes = 701)	701(84.3)	308(43.9)	303(43.2)	63(9.0)	2(0.3)	2(0.3)
Did your pharmacist know how to explain things in an understandable way to you? (Yes = 765)	765(91.9)	364(47.6)	321(42.0)	43(5.6)	3(0.4)	1(0.1)
Did your pharmacist provide information CAM-drug interaction (Yes = 587)	587(70.6)	299(50.9)	230(39.2)	32(5.5)	0(0.0)	1(0.2)

^*^Values in this table represent n (%)

The analysis of the data was carried out for males and females separately and were compared between the two genders. No significant differences were noted in relation to beliefs, perceptions and practices of customers not their satisfaction between males and females in the study population (Additional file 2: Appendix B).

As for the associations of subjects’ characteristics with their beliefs, perceptions and practices towards the role of pharmacists in CAM, the results of the regression analyses showed that, of those characteristics, only age was a significant predictor. More specifically, participants with older age had higher scores for beliefs, perceptions and practices towards the role of pharmacists in CAM (Additional file 3: Appendix C).

## Discussion

This is the first national study to evaluate the customer’s perspectives with regards to the role of Lebanese community pharmacists in orienting and guiding CAM use. The study represents a unique attempt to promote the role of pharmacists in ensuring the safe and effective use of CAM. The findings of this study revealed general positive perception towards the effectiveness of CAM products and of the role of pharmacists coupled with high level of satisfaction from the services delivered by the pharmacists. A few gaps in practices of CAM users and the services they received from the community pharmacists were identified, mainly related the CAM-drug interactions and possible undesirable effect on the health status of the customers. The findings of this study may be used by stakeholders and health authorities to formulate evidence-based recommendations to promote the role of pharmacists (and other health care providers) in ensuring customers’ safety and wellbeing.

Regarding beliefs, the majority of the surveyed customers in this study (84.6%) showed positive beliefs towards the effectiveness of CAM products. Such a positive belief was expected given that the surveyed subjects were identified as users of CAM who bought the CAM product from the pharmacy where they were recruited from. These positive beliefs have been reported in other neighboring countries, such as countries of the Arabian Gulf region, where the positive beliefs towards CAM products were found to be supported by traditional and cultural practices [9–13, 40]. In this study, the majority of customers believed in the safety of CAM with the majority of customers reported that CAM has fewer side effects than conventional medicine. This finding is in accordance with previous literature indicating that the general public usually perceives CAM as safe, possibly due to a common perception that CAM products originated from nature and consequently have no toxic or adverse effect [41, 42]. For instance, cardiac patient from South America reported that CAM provided holistic care, improved the quality of life, and overcame the limitations of conventional medicine. According to these patients, using CAM addressed their need for comprehensive care, and prevented or counteracted adverse effects caused by conventional medicine [42]. Such a misconception of safety exposes consumers to hazardous risks related to CAM side effects, adverse reactions, or drug and food interactions that could cause injury or harm. The safety profile of natural products could sometimes be more complex than conventional medicine. Adverse events associated with CAM products could result from “direct” or “indirect” effect [43]. The direct effect is the intrinsic toxicity related to inherent ingredients in the given product, which is predictable [44]. The indirect effect, on the other hand, is the extrinsic toxicity (contamination, manufacturing problems, adulteration, improper dosage, lack of standardization, etc.), wrong indication, food or drug interaction that can lead to more hazardous non-predictable toxicity [44]. In Lebanon, the implications of these risks could be intensified by the poor regulatory framework of CAM products and the doubts around the standards of their quality [17, 45].

A remarkable finding of this study was the positive perception of the role of pharmacists by CAM consumers. CAM users in Lebanon have high expectations towards the role of pharmacists in CAM. More than 93% of participants perceived that pharmacists should educate them on CAM products’ use and safety coupled with high level of trust (87%) in information from the pharmacists. These findings contrast with previous findings of studies in the region. For instance, the general population in Qatar has a poor understanding of community pharmacists’ role as health care provider [31]. In Kuwait, people had overall negative perceptions of community pharmacists, expressed moderate expectations of their role, and viewed the current pharmacy services as ‘slightly’ positive [46]. Studies from Saudi Arabia reported that the satisfaction, perception and appreciation of the pharmacists’ role in the health care team were improving; however, extra efforts are still needed to improve the skills and perceptions of community pharmacists [38]. The positive perceptions of the role of pharmacists identified in this study represent an opportunity to further promote the role of the pharmacist and to highlight the responsibility of the pharmacists to advice, educate, guide, direct, and persuade the consumer to comply with the proper, safe, and effective use of CAM products.

In this study, the aforementioned positive beliefs and perceptions with regards to the role of pharmacists in this study were found not to be in line with the reported practices. More specifically, one in two surveyed customers did not give feedback to their pharmacists about the outcomes after using CAM. Furthermore, one in five participants did not ask the pharmacists about the safe and effective use of the products nor did they discuss their health status and medication taken before taking CAM products. Such a dichotomy between perceptions and practices is rather alarming: When participants were asked in a theoretical way about whether they perceive the pharmacist to have a role in providing education about the use, side effect, and general health issues, their response tended to be positive. However, when they went to get their product from the pharmacy, they did not discuss the health status or share their feedback or even the outcomes after CAM use. Such a dichotomy has been previously reported, even among patients taking prescribed medications from community pharmacies [47]. In this context, it is important to highlight that the patients’ s feedback constitute the “cornerstone of effective clinical teaching” [48], and can help pharmacists develop their skills and knowledge to better respond to community and customer needs [49]. Patients are often reluctant to volunteer information about their use of herbal products and other “natural” products. Therefore, it is recommended that pharmacists take the initiative and ask their patients about drug history, specially inquiring about the use of CAM, encouraging patients to give them feedback on the outcome or adverse reaction after their use of CAM products. It is through such a rapport with their patients that pharmacists can provide adequate counseling and proper guidance on CAM use [50]. The “*they don’t ask, so I don’t tell*” strategy should be avoided by pharmacists when dispensing CAM products [51].

In this study, older participants had more positive beliefs, perceptions and practices towards the role of pharmacists in CAM, as compared to younger participants. This finding confirms the results of an earlier study in Lebanon investigating the general public’s perceptions towards the community pharmacist’s role. In this report, patients belonging to the older age category reported a higher perception index compared to their younger counterparts [52]. Such an association of age and a better perception of pharmacists’ role has also been reported in studies from other counties such as the United States and Iraq [53, 54]. This association could be explained by the fact that most patients or customers, over time, prefer to visit the same community pharmacy where they feel comfortable. With advancing age, a close patient-pharmacist relationship is developed resulting in a positive perception of the role of the pharmacist’s in one’s health [55]. Another explanation is that, as people have more interactions with the pharmacists, they have a better understanding of his role and are more likely to seek expanded clinical services from their pharmacist [56]. Pharmacists are, hence, encouraged to use this positive perception as an opportunity to make substantive contributions to the health and wellbeing of older patients.

In this study, with regards to the CAM services offered by the pharmacists, the findings showed significant variability. On one hand, the majority of participants reported receiving good education of the CAM product, its side effects and mode of use. Such results are in accordance with a previous investigation about the services of weight management among community pharmacists in Lebanon, and which showed a broad range of services that the pharmacists offered to assist their patients in controlling their weight [24]. Together, these findings further underscore the fact that the pharmacist’s involvement in CAM products is in fact an extension of their roles in pharmaceutical care, clinical pharmacy practices and collaborative healthcare teams’. Professional associations, such as the American College of Clinical Pharmacy (ACCP), the American Society of Health-System Pharmacists and the Canadian Society of Hospital Pharmacists, have recommended that the profession of pharmacy actively embrace dietary supplements (natural health products, vitamins and minerals) as part of the pharmacist’s scope of practice [24, 45]. On the other hand, however, a significant proportion of interviewed CAM users reported that pharmacists were not asking question about their medical history before dispensing CAM products nor were they providing information on CAM-drug interaction. The lack of such services could predispose CAM users to hazardous risks related to possible toxicity or undesirable reactions. These hazardous risks could be exacerbated when it is coupled with safety misconceptions, non-disclosure of use and poor regulatory framework. Possible reasons for failing to provide these services among pharmacists could relate to their dissatisfaction with their working conditions or their limited knowledge in this field. In fact, a previous study in Lebanon showed that pharmacists were not contented with the profit margin, their heavy workload, low number of staff, and long working hours (12–18 h) that are affecting their professional practice and patient counseling [57].

Despite the variability in the proportion of customers receiving proper services related to CAM from the pharmacists, the results of this study indicated that the majority of customers were highly satisfied with the received services. These findings highlighted the fact that when the pharmacists offer a service, they are doing it in an effective manner and the customers are highly satisfied. Such a finding represent an opportunity, especially that community pharmacists are often considered the first contact point between patient and the healthcare system, for which patients have easy and recurrent access [23]. Furthermore, in Lebanon, there exist a large number of active community pharmacist (ratio 7.5 community pharmacy per 10,000 population [57], who can be involved in a broad range of health promotion campaigns and services (other than CAM), including prevention of chronic diseases such as hyperlipidemia, hypercholesterolemia, diabetes, osteoporosis, as well as obesity and weight management [58, 59]. The American Society of Health-System Pharmacists recommends that pharmacists work with their patients to promote behavior modifications towards healthier lifestyles, as they are ideally situated within the community [60].

It is important to note that, in this study, even though participants were recruited from the pharmacies, more than the half of the participants (56.0%) were using CAM as an alternative medicine. This finding is alarming, especially when it is coupled with the fact that 19% of those customers were suffering from chronic diseases. Such a CAM use modality could jeopardize the safety and wellbeing of the patient, more so when the patient does not disclose the use of the CAM to his physician. In fact, previous studies showed very low rate of disclosure from CAM users to healthcare providers for fear of their disapproval, disinterest or inability to help. Such lack of professional supervision may further expose the consumer to various hazardous risks, including drug interactions, adverse reactions or other side effects of the products [6, 61].

Taken together, the findings of this study further underscored the important role that the pharmacist play/can play in ensuring the safe and efficient use of CAM. According to the literature, the main responsibilities for the pharmacist with regards to CAM: acknowledge that CAM is widely used, know the evidence-based recommendations for the common CAMs used among their patients/customers population, ensure that patients are adhering to the safe use of CAM, document the current use of CAM and report its side effects, contribute to heightening the awareness and education about CAM and lastly to collaborate with other health care professionals to ensure a safe and effective use of CAM [27]. Previous studies showed that pharmacists were aware of these important roles in guiding the CAM use, however pharmacists, consistently expressed the necessity for greater access to CAM resources and education opportunities for the safe and effective CAM use [25, 26, 62]. Hence, guidelines are needed to assist pharmacists to making evidence-based decisions in recommending complementary and alternative medicine.

The findings of this study ought to be considered in light of a few limitations. First, although every effort was made to obtain a representative sample from all the Lebanese governorates, the representation of the Beqaa governorate was suboptimal. During the data collection, a travel advisory was issued against going to this part of the country as it was deemed unsafe. Future studies are encouraged to find alternative methods (alternative to physical access) in order to obtain a representative sample of the country including the Beqaa. Second, given that the participants completed the questionnaire during a face-to-face interview with an interviewer, a social desirability bias and an interviewer bias could have affected the findings of this study. That said, all the interviewers in this study were trained on maintaining a neutral attitude and refrain from judgmental hints, hence minimizing any interference with the participants’ responses. Lastly, although the study findings highlighted important issues related to the perspective of CAM users regarding the role of the community pharmacists, the quantitative nature of the study limited an in-depth understanding of the beliefs and the attitude of participants. Future qualitative studies are needed to further explore the aforementioned concepts in further depth and rigor.

## Conclusion

In conclusion, the findings of this study showed that CAM users in Lebanon have positive perceptions and high expectations with regards to the role of pharmacists in guiding their use of CAM use. However such perceptions were not mirrored by the users practices themselves, of who, a significant proportion did not report side effects nor gave feedback to the pharmacists following the use of their CAM. In addition, the study showed that despite their satisfaction with the CAM related services offered by their pharmacists, the users still did not receive critical services such as information about CAM-drug interaction. These findings provide evidence to concerned stakeholders, including public health authorities, educational bodies, and OPL to reflect on the identified gaps and benefits from the opportunities to promote the role of pharmacists in ensuring a safe and effective CAM use Lebanon.

## Supplementary Information

**Additional file 1.** Role of Pharmacists in Complementary and Alternative Medicine: Users’ Perspectives and Satisfaction.

**Additional file 2. **General beliefs with regards to CAM and CAM use among study participants, by gender. (*n* = 832)*.

**Additional file 3.** Predictors of general beliefs with regards to CAM and CAM use among study participants^*^.

## Data Availability

The datasets used and analyzed during the current study are available from the corresponding author on reasonable request.

## Abbreviations

CAM: Complementary and alternative medicine
MENA: Middle East and North Africa
OPL: Order of Pharmacists in Lebanon
SPSS: Statistical Package for Social Sciences
ACCP: American College of Clinical Pharmacy
