# Supplementary material for: The role of pharmacists in complementary and alternative medicine in Lebanon: users’ perspectives
Source: BMC Complement Med Ther. 2021 Mar 2;21:81. doi: 10.1186/s12906-021-03256-8 (PMC7944898; doi:10.1186/s12906-021-03256-8)
Supplement: Supplementary file 1 — Additional file 1. Role of Pharmacists in Complementary and Alternative Medicine: Users’ Perspectives and Satisfaction. [file 12906_2021_3256_MOESM1_ESM.docx]

**Role of Pharmacists in Complementary and Alternative Medicine: Users’ Perspectives and Satisfaction**

Date (dd /mm/ yy): ___/___/___ Subject ID: _______________

**Section A: Socio-demographics**

| - District of the Pharmacy:  1. Beirut 2. South 3. North 4. Mount Lebanon 5. Beqaa 6. Nabatieh | - Age range:_--------------------------------_ - Gender:  1. Male 2. Female  - Why you are using CAM:   ----------------------------------------- | | - Employment status  1. Self-employed 2. Employee 3. Not working 4. Student |
| --- | --- | --- | --- |
| Educational qualification:   1. No education 2. Primary school 3. Higher school 4. Bachelors 5. Higher degree | | - What type of CAM products you are using: - ---------------------------- - How frequent you are using CAM: - ----------------/day - ----------------/week - ----------------/month - ----------------/year | |
| - Do you suffer from any disease:  1. Yes, specify----------------------- 2. No  - Are you using CAM as:  1. Alternative medicine 2. Complementary medicine | | - How you were introduced to CAM  1. Prescription by physicians 2. Through pharmacists 3. Herbalist 4. Self-decision 5. From media 6. Others:---------------------- | |

**Section B: Users’ beliefs towards CAM**

| **Statement** | **1** | **2** | **3** | **4** | **5** |
| --- | --- | --- | --- | --- | --- |
| ***Scale of 1-5 (1 = strongly agree, 2= agree, 3 = neutral, 4 = disagree, 5 = strongly disagree**) | | | | | |
| Do you belief that CAM products are effective |  |  |  |  |  |
| Do you belief that CAM products have less side effect than conventional medicines |  |  |  |  |  |
| Do you think that CAM products available in the Lebanese market are of good quality |  |  |  |  |  |
| Do you think that CAM should be used only for minor diseases |  |  |  |  |  |
| Do you think that CAM can replace medications for chronic and serious diseases |  |  |  |  |  |
| **Section C: Users’ Perception of pharmacists’ role towards CAM** | | | | | |
| Do you think that pharmacist should let you know how to use CAM products and warn you from any possible side effects |  |  |  |  |  |
| Do you think that pharmacist should answer your CAM related questions |  |  |  |  |  |
| Do you trust the pharmacist for the information on the use of CAM products |  |  |  |  |  |
| Do you think that pharmacists should advice customers on general health issues other than about CAM products |  |  |  |  |  |
| Do you think that pharmacists are more expert in CAM products than other healthcare providers? |  |  |  |  |  |

**Section C: Users’ current practice towards CAM within the pharmacies**

| **Statement** | **1** | **2** | **3** | **4** | | **5** | |
| --- | --- | --- | --- | --- | --- | --- | --- |
| ***Scale of 1-5: (1 (always), 2 (often), 3 (sometimes), 4 (rarely), and 5 (no)** | | | | | | | |
| How frequently do you buy your CAM from the pharmacy |  |  |  | |  | |  |
| Do you ask your pharmacist about the effective and safe use of the products |  |  |  | |  | |  |
| Do you give your pharmacist feedback about the outcome after you use CAM |  |  |  | |  | |  |
| Do you give your pharmacist feedback if you suffered from any adverse reaction related to CAM products use? |  |  |  | |  | |  |
| Do you discuss your health status (diseases) and medications taken with your pharmacists before taking CAM products? |  |  |  | |  | |  |

**Section D: Users’ Satisfaction regarding pharmacist’s services in dispensing CAM**

| **Statement** | **1** | **2** | **3** | **4** | **5** |
| --- | --- | --- | --- | --- | --- |
| ***Scale of 1-5 (1 = strongly satisfied, 2= satisfied, 3 = neutral, 4 = dissatisfied, 5 = strongly dissatisfied**) | | | | | |
| Did your pharmacist give you information on the side effect of CAM | - Yes | | - No | | |
| If **Yes**, are you satisfied with the type and amount these information |  |  |  |  |  |
| Did your pharmacist ask you questions about your medical history before dispensing CAM (like disease history, medications, allergy etc.) ? | - Yes | | - No | | |
| If **Yes**, Are you satisfied with these questions? |  |  |  |  |  |
| Did your pharmacist tell you how to use CAM products (dose, timing, duration, etc.) | - Yes | | - No | | |
| If **Yes**, are you satisfied with this information? |  |  |  |  |  |
| Do your pharmacist spend enough time with you when you ask about CAM product? | - Yes | | - No | | |
| If **Yes**, are you satisfied by the amount of time spend by the pharmacist: |  |  |  |  |  |
| Do your pharmacist know how to explain things in an understandable way to you? | - Yes | | - No | | |
| If **Yes**, are you satisfied with the pharmacist’s teaching? |  |  |  |  |  |
| Did your pharmacist provide information CAM-drug interaction. | - Yes | | - No | | |
| If **Yes**, are you satisfied with this information? |  |  |  |  |  |
