# Supplementary material for: The role of pharmacists in complementary and alternative medicine in Lebanon: users’ perspectives
Source: BMC Complement Med Ther. 2021 Mar 2;21:81. doi: 10.1186/s12906-021-03256-8 (PMC7944898; doi:10.1186/s12906-021-03256-8)
Supplement: Supplementary file 2 — Additional file 2. General beliefs with regards to CAM and CAM use among study participants, by gender. (n = 832)*. [file 12906_2021_3256_MOESM2_ESM.docx]

General beliefs with regards to CAM and CAM use among study participants, by gender. (n=832)*

|  | **Strongly agree** | **Agree** | **Neutral** | **Disagree** | **Strongly disagree** | **p-value** |
| --- | --- | --- | --- | --- | --- | --- |
| **Do you belief that CAM products are effective** |  |  |  |  |  |  |
| Overall | 352(42.3) | 352(42.3) | 92(11.1) | 19(2.3) | 17(2.0) | X^2^=2.9  p=0.575 |
| Males | 156(43.3) | 143(39.7) | 46(12.8) | 8(2.2) | 7(1.9) |  |
| Females | 196(41.5) | 209(44.3) | 46(9.7) | 11(2.3) | 10(2.1) |  |
| **Do you belief that CAM products have less side effect than conventional medicines** |  |  |  |  |  |  |
| Overall | 307(36.9) | 327(39.4) | 125(15.0) | 46(5.5) | 26(3.1) | X^2^=2.2  p=0.679 |
| Males | 129(35.9) | 141(39.3) | 59(16.4) | 17(4.7) | 13(3.6) |  |
| Females | 178(37.7) | 186(39.4) | 66(14.0) | 29(6.1) | 13(2.8) |  |
| **Do you think that CAM products available in the Lebanese market are of good quality** |  |  |  |  |  |  |
| Overall | 160(19.3) | 283(34.1) | 235(28.3) | 110(13.3) | 42(5.1) | X^2^=7.4  p=0.116 |
| Males | 84(23.4) | 117(32.6) | 99(27.6) | 42(11.7) | 17(4.7) |  |
| Females | 76(16.1) | 166(35.2) | 136(28.9) | 68(14.4) | 25(5.3) |  |
| **Do you think that CAM should be used only for minor diseases** |  |  |  |  |  |  |
| Overall | 203 (24.4) | 253(30.4) | 208(25.0) | 114(13.7) | 53(6.4) | X^2^=1.0  p=0.917 |
| Males | 87(24.2) | 111(30.9) | 93(25.9) | 48(13.4) | 20(5.6) |  |
| females | 116(24.6) | 142(30.1) | 115(24.4) | 66(14.0) | 33(7.0) |  |
| **Do you think that CAM can replace medications for chronic and serious diseases** |  |  |  |  |  |  |
| Overall | 109(13.1) | 125(15.0) | 186(22.4) | 206(24.8) | 205(24.7) | X^2^=9.7  **p=0.045** |
| Males | 61(17.0) | 56(15.6) | 77(21.4) | 79(22.0) | 86(24.0) |  |
| females | 48(10.2) | 69(14.6) | 109(23.1) | 127(26.9) | 119(25.2) |  |

^*^Values in this table represent n (%). P-values derived from Chi square analysis

Perception of pharmacists’ role in relation to CAM among study participants, by gender. (n=832) ^*^

|  | **Strongly agree** | **Agree** | **Neutral** | **Disagree** | **Strongly disagree** | **p-value** |
| --- | --- | --- | --- | --- | --- | --- |
| **Do you think that the pharmacist should let you know how to use CAM products and warn you for any possible side effects** |  |  |  |  |  |  |
| Overall | 560(67.3) | 219(26.3) | 25(3.0) | 7(0.8) | 21(2.5) | X^2^=2.9  p=0.573 |
| Males | 231(64.2) | 104(28.9) | 12(3.3) | 3(0.8) | 10(2.8) |  |
| females | 329(69.7) | 115(24.4) | 13(2.8) | 4(0.8) | 11(2.3) |  |
| **Do you think that pharmacist should answer your CAM related questions** |  |  |  |  |  |  |
| Overall | 556(67.0) | 223(26.9) | 23(2.8) | 13(1.6) | 15(1.8) | X^2^=3.6  p=0.467 |
| Males | 230(63.9) | 107(29.7) | 10(2.8) | 5(1.4) | 8(2.2) |  |
| females | 326(69.4) | 116(24.7) | 13(2.8) | 8(1.7) | 7(1.5) |  |
| **Do you trust the pharmacist for the information on the use of CAM products** |  |  |  |  |  |  |
| Overall | 434(52.2) | 297(35.7) | 71(8.5) | 17(2.0) | 12(1.4) | X^2^=2.3  p=0.690 |
| Males | 178(49.4) | 134(37.2) | 34(9.4) | 8(2.2) | 6(1.7) |  |
| females | 256(54.4) | 163(34.6) | 37(7.9) | 9(1.9) | 6(1.3) |  |
| **Do you think that pharmacists should advice customers on general health issues other than about CAM products** |  |  |  |  |  |  |
| Overall | 383(46.3) | 283(34.2) | 106(12.8) | 35(4.2) | 20(2.4) | X^2^=5.8  p=0.212 |
| Males | 151(42.1) | 137(38.2) | 46(12.8) | 17(4.7) | 8(2.2) |  |
| females | 232(49.6) | 146(31.2) | 60(12.8) | 18(3.8) | 12(2.6) |  |
| **Do you think that pharmacists are more expert in CAM products than other healthcare providers?** |  |  |  |  |  |  |
| Overall | 172(21.4) | 136(17.0) | 166(20.7) | 143(17.8) | 185(23.1) | X^2^=7.3  p=0.123 |
| Males | 82(23.5) | 58(16.6) | 63(18.1) | 55(15.8) | 91(25.3) |  |
| females | 90(19.9) | 78(17.2) | 103(22.7) | 88(19.4) | 94(20.8) |  |

^*^Values in this table represent n (%). P-values derived from Chi square analysis

Practices of study participants regarding CAM in the pharmacies, by gender. (n=832) ^*^

|  | **n(%)** |  |  |  |  |  |
| --- | --- | --- | --- | --- | --- | --- |
|  | **Always** | **Often** | **Sometimes** | **Rarely** | **No** | **p-value** |
| **How frequently do you buy your CAM products from the pharmacy?** |  |  |  |  |  |  |
| Overall | 501(60.2) | 103(12.4) | 64(7.7) | 79(9.5) | 85(10.2) | X^2^=5.5  p=0.239 |
| Males | 207(57.5) | 44(12.2) | 25(6.9) | 41(11.4) | 43(11.9) |  |
| females | 294(62.3) | 59(12.5) | 39(8.3) | 38(8.1) | 42(8.9) |  |
| **Do you ask your pharmacist about the effective and safe use of the products** |  |  |  |  |  |  |
| Overall | 375(45.1) | 279(33.6) | 94(11.3) | 44(5.3) | 39(4.7) | X^2^=7.0  p=0.137 |
| Males | 157(43.6) | 119(33.1) | 44(12.2) | 16(4.4) | 24(6.7) |  |
| females | 218(46.3) | 160(34.0) | 50(10.6) | 28(5.9) | 15(3.2) |  |
| **Do you give your pharmacist feedback about the outcome after you use CAM** |  |  |  |  |  |  |
| Overall | 217(26.2) | 222(26.8) | 180(21.7) | 85(10.3) | 125(15.1) | X^2^=7.7  p=0.101 |
| Males | 109(30.4) | 93(26.0) | 76(21.2) | 29(8.1) | 51(14.2) |  |
| females | 108(22.9) | 129(27.4) | 104(22.1) | 56(11.9) | 74(15.7) |  |
| **Do you give your pharmacist feedback if you suffered from any adverse reaction related to CAM products use** |  |  |  |  |  |  |
| Overall | 381(45.8) | 263(31.6) | 91(10.9) | 51(6.1) | 46(5.5) | X^2^=4.3  p=0.373 |
| Males | 167(46.4) | 105(29.2) | 41(11.4) | 28(7.8) | 19(5.3) |  |
| females | 214(45.3) | 158(33.5) | 50(10.6) | 23(4.9) | 27(5.7) |  |
| **Do you discuss your health status (diseases) and medications taken with your pharmacists before taking CAM products?** |  |  |  |  |  |  |
| Overall | 332(39.9) | 268(32.2) | 132(15.9) | 48(5.8) | 52(6.3) | X^2^=6.2  p=0.188 |
| Males | 133(36.9) | 111(30.8) | 65(18.1) | 24(6.7) | 27(7.5) |  |
| females | 199(42.2) | 157(33.3) | 67(14.2) | 24(5.1) | 25(5.3) |  |

^*^Values in this table represent n (%). P-values derived from Chi square analysis

Services offered by pharmacists with regards to CAM and their corresponding satisfaction among study participants, by gender. (n=832). ^*^

|  |  | **n(%)** |  |  |  |  |  |
| --- | --- | --- | --- | --- | --- | --- | --- |
|  | **Received the service** | **Of those who received the service** | | | | |  |
|  |  | **Strongly satisfied** | **Satisfied** | **Neutral** | **Dissatisfied** | **Strongly dissatisfied** |  |
| **Did your pharmacist give you information on the side effect of CAM** |  |  |  |  |  |  |  |
| Overall | 725(87.1) | 342(47.2) | 305(42.1) | 43(5.9) | 6(0.8) | 0(0.0) | X^2^=2.3  p=0.512 |
| Males | 303(84.2) | 141(39.2) | 134(37.2) | 14(3.9) | 2(0.6) | 0(0.0) |  |
| females | 422(89.4) | 201(42.6) | 171(36.2) | 29(6.1) | 4(0.8) | 0(0.0) |  |
| **Did your pharmacist ask you questions about your medical history before dispensing CAM (like disease history, medications, allergy etc.)?** |  |  |  |  |  |  |  |
| Overall | 651(78.2) | 327(50.2) | 263(40.4) | 33(5.1) | 3(0.5) | 1(0.2) | X^2^=4.1  p=0.389 |
| Males | 274(76.3) | 146(40.6) | 104(28.9) | 12(3.3) | 2(0.6) | 1(0.3) |  |
| females | 377(80) | 181(38.2) | 159(33.7) | 21(4.4) | 1(0.2) | 0(0.0) |  |
| **Did your pharmacist tell you how to use CAM products (dose, timing, duration, etc.)** |  |  |  |  |  |  |  |
| Overall | 777(93.4) | 411(52.9) | 301(38.7) | 29(3.7) | 2(0.3) | 1(0.1) | X^2^=4.0  p=0.412 |
| Males | 328(91.1) | 173(48.1) | 134(37.2) | 8(2.2) | 1(0.3) | 0(0.0) |  |
| females | 449(95.1) | 238(50.4) | 167(35.4) | 21(4.4) | 1(0.2) | 1(0.2) |  |
| **Did your pharmacist spend enough time with you when you ask about CAM product?** |  |  |  |  |  |  |  |
| Overall | 701(84.3) | 308(43.9) | 303(43.2) | 63(9.0) | 2(0.3) | 2(0.3) | X^2^=2.1  p=0.719 |
| Males | 239(81.6) | 133(36.9) | 126(35.0) | 24(6.7) | 0(0.0) | 1(0.3) |  |
| females | 408(86.4) | 175(37.1) | 177(37.5) | 39(8.3) | 2(0.4) | 1(0.2) |  |
| **Did your pharmacist know how to explain things in an understandable way to you?** |  |  |  |  |  |  |  |
| Overall | 765(91.9) | 364(47.6) | 321(42.0) | 43(5.6) | 3(0.4) | 1(0.1) | X^2^=3.1  p=0.544 |
| Males | 326(91.6) | 147(40.8) | 145(40.3) | 19(5.3) | 2(0.6) | 0(0.0) |  |
| females | 439(93.8) | 217(46.0) | 176(37.3) | 24(5.1) | 1(0.2) | 1(0.2) |  |
| **Did your pharmacist provide information CAM-drug interaction** |  |  |  |  |  |  |  |
| Overall | 587(70.6) | 299(50.9) | 230(39.2) | 32(5.5) | 0(0.0) | 1(0.2) | X^2^=2.7  p=0.435 |
| Males | 253(70.7) | 138(38.3) | 98(27.2) | 11(3.1) | 0(0.0) | 0(0.0) |  |
| females | 334(71.1) | 161(34.1) | 132(28.0) | 21(4.4) | 0(0.0) | 1(0.2) |  |

^*^Values in this table represent n (%). P-values derived from Chi square analysis
