# Supplementary material for: The role of pharmacists in complementary and alternative medicine in Lebanon: users’ perspectives
Source: BMC Complement Med Ther. 2021 Mar 2;21:81. doi: 10.1186/s12906-021-03256-8 (PMC7944898; doi:10.1186/s12906-021-03256-8)
Supplement: Supplementary file 3 — Additional file 3. Predictors of general beliefs with regards to CAM and CAM use among study participants*. [file 12906_2021_3256_MOESM3_ESM.docx]

Predictors of general beliefs with regards to CAM and CAM use among study participants^*^

|  | **B, 95% CI** | **Adjusted B, 95% CI^**^** |
| --- | --- | --- |
| **Age (years)** |  |  |
| 18-25 | Ref | Ref |
| 26-33 | **0.082(0.008,0.485)** | 0.058(-0.086,0.439) |
| 34-50 | **0.129(0.129,0.560)** | **0.111(0.057,0.542)** |
| ≥51 | **0.127(0.154,0.648)** | **0.112(0.117,0.656)** |
| **Gender** |  |  |
| Male | Ref | - |
| Female | -0.032(-0.249,0.091) | - |
| **Employments status** |  |  |
| Self-employed | Ref | Ref |
| Employee | -0.028(-0.340,0.201) | 0.003(-0.270, 0.284) |
| Not working/student | -0.090(-497, 0.047) | -0.033(-0.370, 0.205) |
| **Highest educational level attained** | | |
| No education/ Primary school | Ref | Ref |
| High school | **-0.116(-0.641, -0.059)** | -0.081(-0.549,0.060) |
| Bachelors/ Higher degrees | -0.070(-0.429,0.068**)** | -0.033(-0.360,0.187) |
| **Do you suffer from any disease** | | |
| No | Ref | - |
| Yes | 0.020(-0.135, 0.248) | - |

^*^ Numbers in this table represent the linear regression coefficients β and their corresponding 95% CI interval

^**^Variables that were significantly associated with the outcome (p-value< 0.2) in the simple regression analyses were entered in the multiple regression model

Predictors of perception of pharmacists’ role in relation to CAM among study participants^*^

|  | **B, 95% CI** | **Adjusted B, 95% CI**^**^ |
| --- | --- | --- |
| **Age range** |  |  |
| 18-25 | Ref | Ref |
| 26-33 | 0.049(-0.101,0.432) | 0.054(-0.086,0.447) |
| 34-50 | **0.128(0.141,0.623)** | **0.133(0.154,0.636)** |
| ≥51 | **0.082(0.015,0.568)** | **0.093(-0.049,0.607)** |
| **Gender** |  |  |
| Male | Ref | Ref |
| Female | 0.049(-0.052, 0.326) | 0.061(-0.022,0.359) |
| **Employments status** |  |  |
| Self-employed | Ref | - |
| Employee | 0.029(-0.220,0.380) | - |
| Not working/student | -0.037(-0.406,0.199) | - |
| **Highest educational level attained** | | |
| No education/ Primary school | Ref | - |
| High school | 0.030(-0.226,0.429) | - |
| Bachelors/ Higher degrees | -0.006(-0.298,0.262) | - |
| **Do you suffer from any disease** | | |
| No | Ref | - |
| Yes | 0.042(-0.085,0.347) | - |

^*^ Numbers in this table represent the linear regression coefficients β and their corresponding 95% CI interval

^**^Variables that were significantly associated with the outcome (p-value< 0.2) in the simple regression analyses were entered in the multiple regression model

Predictors of practices of study participants regarding CAM in the pharmacies^*^

|  | **B, 95% CI** | **Adjusted B, 95% CI**^**^ |
| --- | --- | --- |
| **Age range** |  |  |
| 18-25 | Ref | Ref |
| 26-33 | 0.061(-0.049,0.352) | -0.061(-0.049,0.352) |
| 34-50 | **0.122(0.087,0.451)** | **0.122(0.087,0.451)** |
| ≥51 | 0.043(-0.097,0.322) | **0.043(-0.097,0.322)** |
| **Gender** |  |  |
| Male | Ref | - |
| Female | 0.000(-0.142,0.143) | - |
| **Employments status** |  |  |
| Self-employed | Ref | - |
| Employee | -0.011(-0.248,0.202) | - |
| Not working/student | -0.042(-0.314,0.139) | - |
| **Highest educational level attained** | | |
| No education/ Primary school | Ref | - |
| High school | -0.073(-0.430,0.064) | - |
| Bachelors/ Higher degrees | -0.001(-0.208,0.214) | - |
| **Do you suffer from any disease** | | |
| No | Ref | - |
| Yes | 0.015(-0.128, 0.200) | - |

^*^ Numbers in this table represent the linear regression coefficients β and their corresponding 95% CI interval

^**^Variables that were significantly associated with the outcome (p-value< 0.2) in the simple regression analyses were entered in the multiple regression model
